# The Effects on Liver Metastases of Circadian Patterned Continuous Hepatic Artery Infusion of FUDR

**DOI:** 10.1155/1994/54058

**Published:** 1994

**Authors:** M. Margaret Kemeny, Galo Alava, Jorge M. Oliver

**Affiliations:** 1 Department of Surgery and Pathology North Shore University Hospital Manhasset NY 11030 USA; 2 Surgical Oncology St. Vincent's Hospital 153 West 11th St. New York 10011 USA

## Abstract

Although continuous hepatic artery infusions (CHAI) of (FUDR) Floxuridine have been effective in reducing the size of colorectal hepatic metastases the toxicity of the infusions have been high with almost a quarter of the patients developing biliary sclerosis. Techniques to lower toxicity, yet continue the beneficial antitumor effects, are being investigated. One suggested strategy is to change the flow pattern of the continuous infusion from a constant rate to a day cycled pattern. In this infusion a continuous rate is given over a 24 hour period with 60% of the infusion delivered between 3 PM and 9 PM and the least amount of infusion delivered between 3 AM and 9 PM. Previous research has suggested that this day cycle pattern will lower the toxicity of the infusion. This experiment is a test of “day cycled” continuous hepatic artery infusions in rats bearing hepatic metastases from a colon adenocarcinoma. Previous research from our laboratory has shown a lowered toxicity when the constant infusion was replaced with the day cycled pattern.

In the present study 10 rats with hepatic adenocarcinoma metastases were placed on constant CHAI of FUDR at 10mg/kg/day for 14 days. There was an 80% mortality from chemotherapy toxicity and a 90% objective response rate. Nine other rats were treated with “day cycled” CHAI of FUDR at 15mg/kg/d. There was no mortality in this group and the objective response rate was 90% as in the previous group. This study demonstrated that “day cycled” CHAI of FUDR was substantially less toxic and that the antitumor effect was identical to the constant infusion.


*HPB Surgery*, 1994, Vol. 7, pp. 219–224
Reprints available directly from the publisher
Photocopying permitted by license only

© 1994 Harwood Academic Publishers GmbH
Printed in the United States of America

### THE EFFECTS ON LIVER METASTASES OF CIRCADIAN PATTERNED CONTINUOUS HEPATIC ARTERY INFUSION OF FUDR

M. MARGARET KEMENY, GALO ALAVA and JORGE M. OLIVER
*Department of Surgery and Pathology, North Shore University Hospital, Manhasset, NY 11030*

*(Received 6 October 1992)*

Although continuous hepatic artery infusions (CHAI) of (FUDR) Floxuridine have been effective in reducing the size of colorectal hepatic metastases the toxicity of the infusions have been high with almost a quarter of the patients developing biliary sclerosis. Techniques to lower toxicity, yet continue the beneficial antitumor effects, are being investigated. One suggested strategy is to change the flow pattern of the continuous infusion from a constant rate to a day cycled pattern. In this infusion a continuous rate is given over a 24 hour period with 60% of the infusion delivered between 3 PM and 9 PM and the least amount of infusion delivered between 3 AM and 9 PM. Previous research has suggested that this day cycle pattern will lower the toxicity of the infusion. This experiment is a test of "day cycled" continuous hepatic artery infusions in rats bearing hepatic metastases from a colon adenocarcinoma. Previous research from our laboratory has shown a lowered toxicity when the constant infusion was replaced with the day cycled pattern.

In the present study 10 rats with hepatic adenocarcinoma metastases were placed on constant CHAI of FUDR at 10mg/kg/day for 14 days. There was an 80% mortality from chemotherapy toxicity and a 90% objective response rate. Nine other rats were treated with "day cycled" CHAI of FUDR at 15mg/kg/d. There was no mortality in this group and the objective response rate was 90% as in the previous group. This study demonstrated that "day cycled" CHAI of FUDR was substantially less toxic and that the antitumor effect was identical to the constant infusion.

KEY WORDS: Circadian rhythm, hepatic artery infusion

### INTRODUCTION

Continuous hepatic artery infusions (CHAI) of floxuridine (FUDR) through an implantable pump was popularized as a treatment for hepatic metastases from colorectal cancer in the early 1980s. Although there was a wave of initial enthusiasm, this began to die down as it became clear that there were substantial toxicities that accompanied the significant benefits seen with this mode of therapy.

The most serious of the toxicities, biliary sclerosis, also known as sclerosing cholangitis, is a fibrosis of the bile ducts, usually at the hepatic duct bifurcation, seen mainly in patients who were responders to therapy[1]. Several modifications of the treatment have been tried to see if toxicity could be reduced, including a reduction of the dose of chemotherapy and the addition of Decadron to the infusion[2,3]. Both of these modifications helped somewhat but didn't get rid of the problem.

Address correspondence to: M. Margaret Kemeny, M.D., F.A.C.S., Chief, Surgical Oncology, St. Vincent's Hospital, 153 West 11th St., New York 10011, USA.

Recently interest has grown for the idea of circadian patterning of the continuous infusion which might reduce the toxic effect of chemotherapy on normal liver and have an equal effect on the tumor cells as the constant continuous infusion. These hypotheses have not been tested in an experimental model using hepatic artery infusion. This experiment is a test in a rat model of the efficacy of CHAI of floxuridine (FUDR) with circadian patterning against established liver metastases.

### MATERIALS AND METHODS

### *Animals*

Fisher rats were kept in the St. Vincent's Medical Center animal facility. They were fed ad lib on a diet of rat chow. The lighting was set to a routine of 8 a.m. to 8 p.m. lights on and 8 p.m. to 8 a.m. lights off.

### *Tumor*

A colon adenocarcinoma line (dinitrobenzene induced colon cancers purchased from the National Cancer Institute) was passaged in Fisher rats using a subcutaneous injection into the hind quarter of animals. When the tumor was palpable and over 3 cm in size it was felt to be ready for use in the experiments. Rats were sacrificed and the tumor was resected surgically. It was then sterilely minced into small pieces and passed through a number 40 screen. The tumor was then placed in RPMI media with streptomycin and penicillin and washed three times. Tumor was resuspended in Hanks Basic Salt Solution to a volume of 1 cc. This slurry of cells was then used for injection ($1 \times 10^7$ cells/cc). The rats were given general anesthesia with use of Ketamine (Ketalar-Parke Davis) 45 mg/kg mixed with Droperidol 0.1 mg/kg by intra muscular injection. A right lateral subcostal incision was used to enter the peritoneum. The right lobe of the liver was pulled out into the wound and a subcapsular injection of 0.1cc of the cell slurry mentioned above was injected. A swab of collodium was placed over the injection site to seal the needle puncture wound. The liver was then pushed back into the peritoneum and the abdomen was closed with staples. Ten days after implantation of tumor into the liver a midline laparotomy was done. At the time of this laparotomy the liver tumor was measured. An established tumor had to be measurable in 3 dimensions for the rat to be used for the experiment.

### *Placement of the Arterial Catheter*

Ten days after tumor implantation each rat underwent a laparotomy for placement of a PE-10 Polyethylene tube (Clay Adams) for hepatic artery infusion. At that time the liver tumor was measured before going ahead with the catheterization. The average rat was 250 gm at the time of the operation and eight weeks old. The catheter was placed into the gastroduodenal artery and the artery was ligated distally. The catheter was then passed through the abdominal fascia, tunnelled under the skin to an opening between the scapulae. At that spot a metal button was placed subcutaneously with a coiled metal catheter attached (Instatech). The PE catheter was passed through the button into the coiled metal tube and was attached to a swivel placed above the rat's cage. This swivel was connected to the infusion pump. This arrangement allowed the rat free mobility in its cage, as well as protecting the button apparatus. For additional protection a small jacket was placed on the rat, preventing it from chewing on the catheter exit site.

The portable Intelliject pumps were used for the infusions because of their programmability for a varied infusion cycle. The accuracy of flow rates were verified before use.

Ten animals were placed on a constant infusion cycles and 9 on a "day cycled" infusion. The infusions were continuous for 14 days. After the 14 day infusion heparinized saline was infused for an additional 14 days. Then the animals were sacrificed, their livers were removed, the hepatic tumors were measured in 3 dimensions and the livers were sent to pathology.

*Infusion Schedules*

Rats given the constant CHAI received 4cc/day of FUDR (Roche) at 10 mg/kg/day. Rats on the "day cycle" infusion received 4cc/day of FUDR at 15mg/kg/day. The delivery dose was as follows (Figure 1):

From 9AM to 3PM 0.6 cc (.1cc/hr)
From 3PM to 9PM 2.7 cc (.45cc/hr)
From 9PM to 3AM 0.6 cc and
From 3AM to 9AM 0.08 cc (.013 cc/hr).

All experiments were approved by the Animal Welfare Committee of St. Vincent's Hospital in accordance to the guidelines of the Public Health Service Policy of Humane Care and Use of Laboratory Animals.

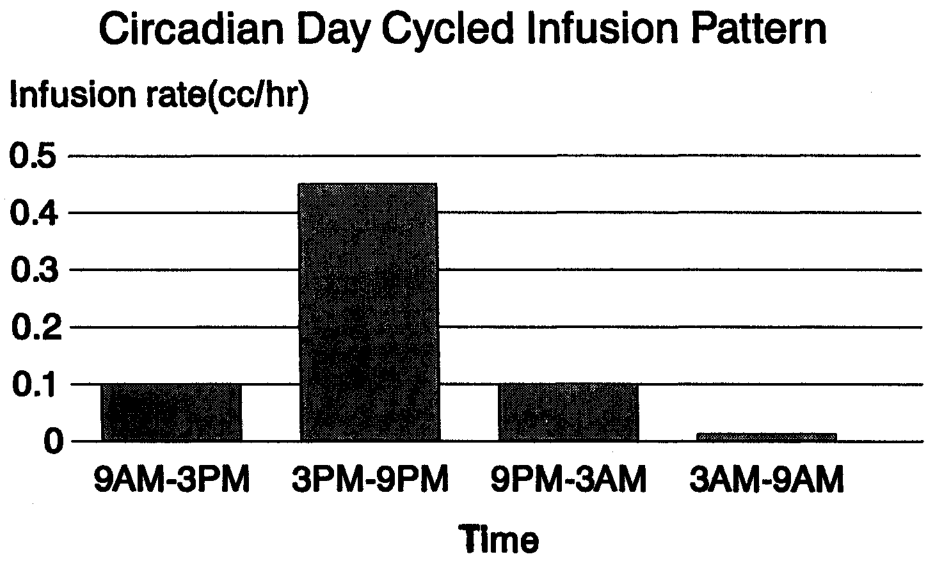

### RESULTS

Ten rats were given a constant FUDR infusion of 10 mg/kg/d for 14 days. Infusions were started 10 days after hepatic implantation of adenocarcinoma. The mean tumor volume at the time of laparotomy for catheter placement was 1022 ± 138mm$^3$. All 10 animals had a decrease in tumor volume as a result of the infusion. The post infusion mean volume was 68.2 ± 26.6 mm$^3$. Eight of the animals died of infusion toxicity prior to the end of the 30 day period. The 2 remaining animals survived the 30 day period. The mean survival for the 8 animals who died was 14.95 ± 4.74 days.

Nine rats were given a CHAI of FUDR with a day cycle circadian pattern at 15 mg/kg/d. All of these animals survived the 14 day infusion and 14 day rest period. Eight rats had a reduction in tumor and one had no change. The mean tumor volume pre infusion was 1202 ± 371 mm$^3$ and the mean post infusion volume was 34.78 ± 19.8 mm$^3$. There was no statistically significant difference in response between the constant infusion group or the circadian patterned infusion group (Figure 2).

### PATHOLOGY

The livers were fixed in 10% buffered formalin. All livers were sectioned at 3 mm intervals and no less than 2 blocks were submitted per case for paraffin sections. A minimum 4 cm$^2$ of cross sectional area per case was examined using standard hematoxylin/eosin staining.

The livers from the rats with the constant infusion FUDR contained necrotic tumor in most cases with focal but widespread centrilobular necrosis of the hepatic parenchyma. The specimens from the animals treated with the circadian rhythm patterns had limited focal necrosis of the hepatic parenchyma. The tumors were necrotic and similar in appearance to the constant infusion group.

### DISCUSSION

The use of circadian rhythm for timing the infusions of chemotherapy is gaining acceptance with some of the recent studies on intravenous chemotherapy. A study reported this year at ASCO cited a 58% response rate in patients who had metastases from colorectal cancer when their intravenous chemotherapy was modulated by a circadian rhythm pattern[4]. This response rate was even more remarkable since most of the patients had failed other chemotherapy regimens.

Other studies have shown that in rats a continuous FUDR intravenous infusion is given with the majority of the daily infusion delivered from 3PM to 9PM and the least delivered from 3AM to 9AM is less toxic than a constant continuous infusion[5]. The concept of decreased toxicity with a "day cycle" circadian patterned continuous infusion has been utilized clinically for CHAI of FUDR for hepatic metastases from colorectal primaries[5]. These studies suggest that toxicity is lowered by using a circadian timed infusion and that antitumor efficacy is not compromised.

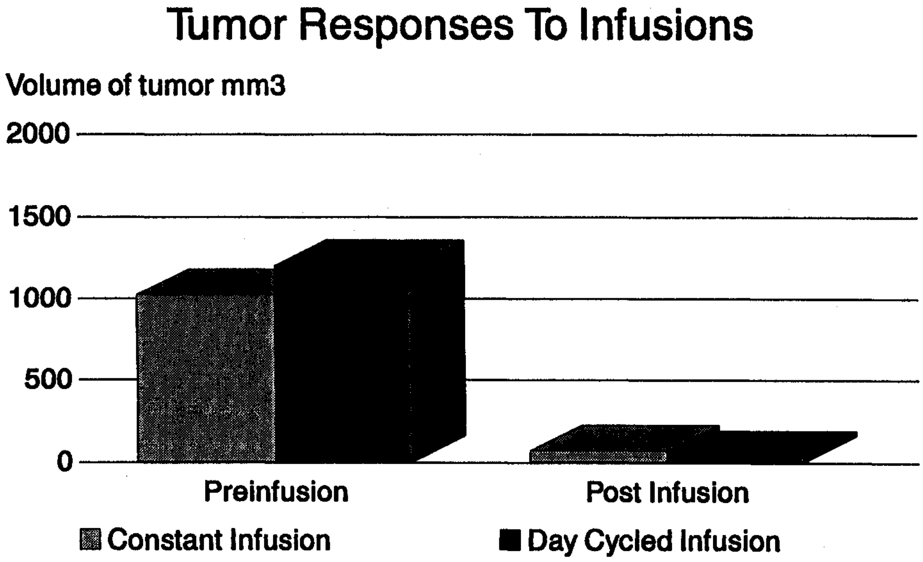

In our laboratory we have developed an animal model that utilizes continuous hepatic artery infusions (CHAI) for prolonged periods of time (one month or longer) in rats. Using this model we have previously reported on toxicity experiments with CHAI of FUDR[6]. In the previously done experiments rats given constant CHAI of FUDR at 15 mg/kg/d all died at 9.3 days ± 1.7 SD. The rats given a circadian patterned CHAI of FUDR at 15 mg/kg/d, with the majority of the daily dose given between 3PM and 9PM (what we call a "day cycle" pattern), all survived the infusion with minimal pathological hepatic toxicity. When the infusion was given with a "night cycle" pattern, that is most of the infusion was given between 9PM and 3AM, it was more toxic with all animals dying at a mean of 5.5 days ± 1.2 SD.

Using these results we went ahead to test the efficacy of circadian infusions against established metastatic colon adenocarcinomas transplanted into the liver. In these present experiments we have shown that day cycled infusions allow more FUDR to be given with lower toxicity and an equal antitumor effect. More specifically 15 mg/kg/d of FUDR was administered for a 14 day CHAI with no toxicity versus an 80% toxicity when 10 mg/kg/day were given using a constant CHAI pattern.

The timing of the "day cycle" was picked because of the previous work by Hrushevsky. The mechanism for the decreased toxicity is unknown but various hypotheses have been proposed. It is known that when 5FU is given with a continuous intravenous infusion the concentration of the drug in the blood varies with time during a 24 hour period[7]. It was discovered that the highest concentrations were at night. The mechanism for FU metabolism is mainly centered in the liver. Thus it seems that hepatic metabolism of this drug is higher during the day. This increase in hepatic metabolism during daylight hours may explain the results of the present experiment. With increased metabolism of the drug during the time of greatest exposure (during the day) the systemic toxicities would be decreased. Because it is a regional infusion the tumor cells are being exposed to the FUDR at higher doses before it has been metabolized so the antitumor effect is not compromised by the increased metabolism.

Other variations known to occur with a circadian pattern include increased corticosteroid levels in the late afternoon (in rats)[8], increased T lymphocyte release (at night), increased CD4 cell activation (in afternoon), and increased antigen recognition and destruction (in the morning)[9]. These changes might also effect toxicity and tumor response in a circadian pattern.

Further work should be done to ascertain if the present "day cycle" circadian schedule is the best one for decreasing toxicity and if it is also the optimal one for antitumor effect. When these criteria have been worked out this modality will be ready to enter the clinical arena.

### *References*

1. Kemeny, M.M., Battifora, H., Blayney, D.W. *et al.* (1985) Sclerosing Cholangitis after Continuous Hepatic Artery Infusion of FUDR. *Ann. Surg.*, **202**, 176–181
2. Hohn, D.C., Stagg, R.J., Friedman, J.F. *et al.* (1989) A randomized trial of continuous intravenous versus hepatic intraarterial floxuridine in patients with colorectal cancer metastatic to the liver: The Northern California Oncology Group Trial. *J. Clin. Onc.*, **11**, 1646–1654
3. Kemeny, N., Seiter, K., Niedzwiecki, D. *et al.* (1992) A randomized trial of intrahepatic infusion of fluorodeoxyuridine with dexamethasone versus fluorodeoxyuridine alone in the treatment of metastatic colorectal cancer. *Cancer*, **69**, 327–334
4. Levi, F., Brienza, S., Misset, J.L. *et al.* (1992) Circumvention of clinical resistance of metastatic colorectal cancer to 5-fluorouracil (5-FU) with circadian rhythm modulated venous chemotherapy. *Proceeding of ASCO*, **11**, 500
5. Roemeling, R.V. and Hrushesky, W.J.M. (1987) Circadian pattern of continuous FUDR infusion reduces toxicities. *Advances in Chronobiology*, Part B, 357–373
6. Kemeny, M.M., Alava, G., Oliver, J.M. and Smith, F.B. (1992) The effects on toxicity of circadian patterning of continuous hepatic artery infusion. *HPB Surg.*, **5**, 185–194
7. Petit, E., Milano, G., Levi, F. *et al.* (1988) Circadian rhythm-varying plasma concetration of 5-fluorouracil during a five-day continuous venous infusion at a constant rate in cancer patients. *Cancer Research*, **48**, 1676–1679
8. Loeb, W.F. and Quimby, F.W. (1989) *The clinical chemistry of laboratory animals*, p. 292, Pergamon Press
9. Levi, F., Canon, C., Dipalma, M. *et al.* (1991) When should the immune clock be reset? From circadian pharmacodynamics to temporally optimized drug delivery. *Annals of the Academy of Sciences*, **618**, 312–329

*(Accepted by S. Bengmark 24 April 1993)*

